# Triglyceride-glucose waist-to-height ratio in relation to prevalent and incident multimorbidity: cross-sectional and prospective evidence from four national studies

**DOI:** 10.1186/s12933-026-03320-y

**Published:** 2026-08-24

**Authors:** Zhengqi Shao, HongZhi Hu, Xing Ge, Mingjie Liu, Yuting Duan, Xiangru Guo, Danqi Zhu, Zixuan Yi, Bai Wei

**Affiliations:** 1 Department of Oncology, Liyuan Hospital, Tongji Medical College, Huazhong University of Science and Technology, Wuhan, 430077 Hubei China; 2 Department of Orthopaedics, Union Hospital, Tongji Medical College, Huazhong University of Science and Technology, Wuhan, 430022 China

**Keywords:** TyG-WHtR index, Multimorbidity, Insulin resistance, Central obesity, Threshold effect, CHARLS, ELSA, NHANES, HRS

## Abstract

**Background:**

The triglyceride-glucose waist-to-height ratio (TyG-WHtR) integrates insulin resistance and central adiposity, but its association with generalized multimorbidity across populations and its dose–response threshold remain uncertain.

**Methods:**

We analyzed adults aged ≥ 50 years from five analytic samples derived from four national studies. Cross-sectional analyses included the National Health and Nutrition Examination Survey (NHANES; n = 1842) and the Health and Retirement Study (HRS; n = 4310), whereas prospective analyses included the China Health and Retirement Longitudinal Study (CHARLS; n = 4114), the English Longitudinal Study of Ageing (ELSA; n = 1310), and HRS (n = 971). Multimorbidity was defined as the presence of at least two of eight harmonized chronic conditions. Multivariable logistic regression and Cox proportional hazards models were used to evaluate prevalent and incident multimorbidity, respectively. Restricted cubic spline and two-piecewise regression models were used to assess nonlinear associations, and receiver operating characteristic curves were used to compare TyG-WHtR with five alternative TyG-related anthropometric indices.

**Results:**

In the cross-sectional analyses, the fully adjusted ORs comparing the highest with the lowest TyG-WHtR quartile were 5.04 (95% CI 3.02–8.42) in NHANES and 3.63 (95% CI 2.70–4.90) in HRS. In the prospective analyses, the fully adjusted HRs comparing the highest with the lowest quartile were 1.87 (95% CI 1.59–2.21) in CHARLS, 1.67 (95% CI 1.30–2.15) in ELSA, and 1.57 (95% CI 1.08–2.29) in HRS. Prospective dose–response associations were nonlinear, with inflection points of 5.92, 5.80, and 6.69, respectively; risk increased predominantly below these values. TyG-WHtR had the largest area under the curve in both cross-sectional samples and in CHARLS and ELSA. Lagged analyses and alternative multimorbidity definitions yielded consistent results.

**Conclusions:**

Higher TyG-WHtR was associated with prevalent multimorbidity in both cross-sectional samples and with incident multimorbidity in quartile-based analyses across all three prospective cohorts, although the continuous association was not statistically significant in prospective HRS. The cohort-specific inflection points suggest a provisional candidate risk-alert interval of approximately 5–7. This interval requires independent external validation and should not be interpreted as a diagnostic or treatment threshold.

**Graphical abstract:**

**Figure:**
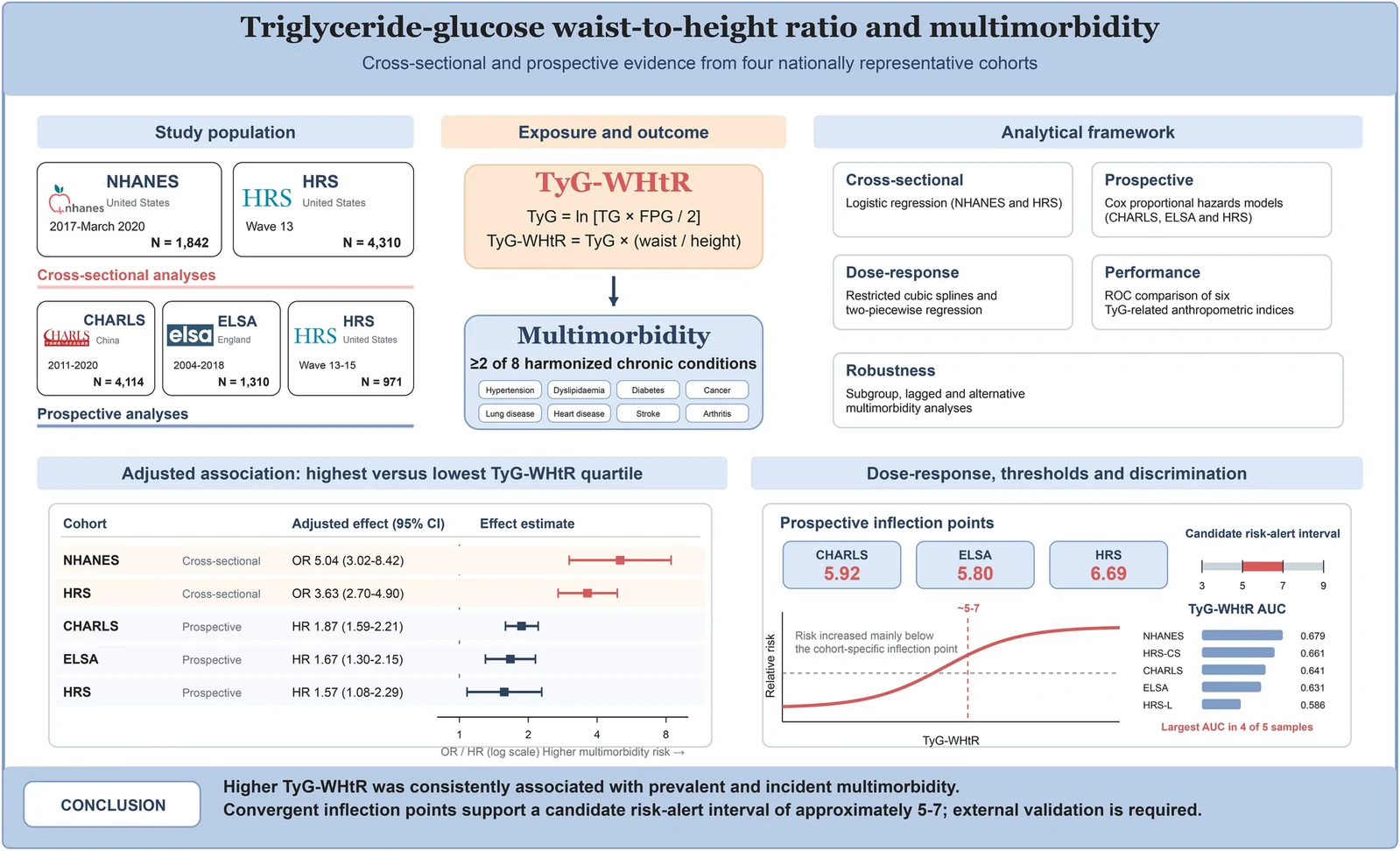

**Supplementary Information:**

The online version contains supplementary material available at https://doi.org/10.1186/s12933-026-03320-y.

## Introduction

Multimorbidity, defined as the presence of two or more chronic diseases, represents a significant health threat, particularly among middle-aged and older adults [1]. Globally, the prevalence of multimorbidity is estimated at 37.2%, though this figure varies considerably by region [2]. Continent-level data reveal that South America has the highest rate (47.5%), followed by North America (43.1%), Europe (39.2%), and Asia (35%) [3]. In China, a nationwide cohort study reported that the prevalence among adults aged ≥ 50 years reached 42.4%, escalating to 61% among those aged ≥ 70 years [4, 5].The high prevalence of multimorbidity leads to several adverse outcomes, such as elevated mortality rates, greater healthcare utilization, and higher medical expenses [3]. A recent meta-analysis showed that individuals with at least two chronic conditions had a 1.73-fold higher risk of death than those without multimorbidity [6].This burden is compounded by aging populations, which drive a continual escalation in healthcare demands and costs for multimorbidity [7, 8].Given the rising prevalence and substantial health risks associated with multimorbidity, early detection and targeted interventions are urgently needed to prevent adverse clinical outcomes.

Accumulating evidence indicates that individuals with multimorbidity exhibit significantly higher levels of circulating inflammatory biomarkers compared to their healthy counterparts [9].This chronic, low-grade systemic inflammation has been increasingly recognized as a core pathophysiological driver that promotes the development of insulin resistance (IR) and metabolic dysfunction [10]. IR refers to the impaired regulation of insulin-mediated glucose metabolism in target tissues [11]. While the insulin-normoglycemic clamp is the gold standard for assessing IR status, its invasive, expensive, and complex nature makes it impractical for routine clinical monitoring [12].The triglyceride-glucose (TyG) index has gained recognition as a feasible and economical surrogate biomarker for assessing IR [13], and it has been implicated in the pathogenesis of numerous chronic conditions, including cardiovascular diseases (e.g., coronary heart disease [14] and ischemic stroke [15]), metabolic disorders (e.g., diabetes mellitus [16]), and other systemic diseases such as hepatic, renal, and malignant pathologies [17–19].

Characterized by IR and lipid dysregulation, the global obesity pandemic has exacerbated the burden of age-related chronic diseases [20]. Anthropometric measures such as waist circumference (WC), waist-to-hip ratio (WHR), and waist-to-height ratio (WHtR) are commonly used to assess it [21]. Notably, WHtR provides a height-normalized assessment that more effectively distinguishes visceral adipose tissue (VAT) from subcutaneous adipose tissue, offering a standardized evaluation of central obesity [22]. Because the TyG index primarily reflects disturbances in glucose and lipid metabolism, whereas anthropometric indices characterize adiposity and body-fat distribution, combining TyG with these measures may provide a more comprehensive assessment of metabolic burden than either component alone [23].

Accordingly, several TyG-related adiposity indices have been developed, including TyG-BMI, TyG-WC, TyG-WHtR, TyG-body roundness index (TyG-BRI), TyG-a body shape index (TyG-ABSI), and TyG-weight-adjusted waist index (TyG-WWI). These indices are not interchangeable: BMI primarily reflects general adiposity, WC reflects abdominal size, WHtR standardizes abdominal adiposity for height, whereas BRI, ABSI, and WWI attempt to characterize body shape or central fat accumulation while reducing the influence of overall body size [24, 25]. Emerging evidence suggests that TyG-related composite indices may improve cardiometabolic risk stratification compared with TyG alone [24–26]. However, their relative performance varies according to the study population and clinical outcome, and no single index has been consistently established as optimal.

Despite growing evidence linking TyG-WHtR and other TyG-related indices to cardiovascular disease, diabetes, cancer, and mortality [21, 27, 28], important knowledge gaps remain. First, most previous studies have focused on individual diseases or cardiometabolic multimorbidity rather than generalised multimorbidity involving multiple organ systems [29, 30]. Second, available evidence has largely been derived from single-country or predominantly East Asian populations, leaving cross-population generalisability uncertain. Furthermore, heterogeneity in the number and composition of chronic conditions across cohorts may compromise the comparability of multimorbidity estimates. Therefore, it remains unclear whether TyG-WHtR is consistently associated with generalised multimorbidity across diverse populations and whether it offers superior or complementary discriminatory value compared with other TyG-related indices.

Accordingly, this study used five analytic samples from four nationally representative studies to examine the cross-sectional and prospective associations between TyG-WHtR and harmonized multimorbidity among adults aged ≥ 50 years. We further investigated nonlinear and threshold associations, compared TyG-WHtR with five alternative TyG-related anthropometric indices, and assessed the robustness of the findings through subgroup and sensitivity analyses.

## Methods

### Study design and study populations

This multicohort study used data from four large, nationally representative population-based studies and comprised five analytic samples. Cross-sectional analyses were conducted using the National Health and Nutrition Examination Survey (NHANES) and the Health and Retirement Study (HRS), whereas prospective analyses were conducted using the China Health and Retirement Longitudinal Study (CHARLS), the English Longitudinal Study of Ageing (ELSA), and HRS. To improve comparability across databases, all analyses were restricted to participants aged 50 years or older.

**Fig. 1:**
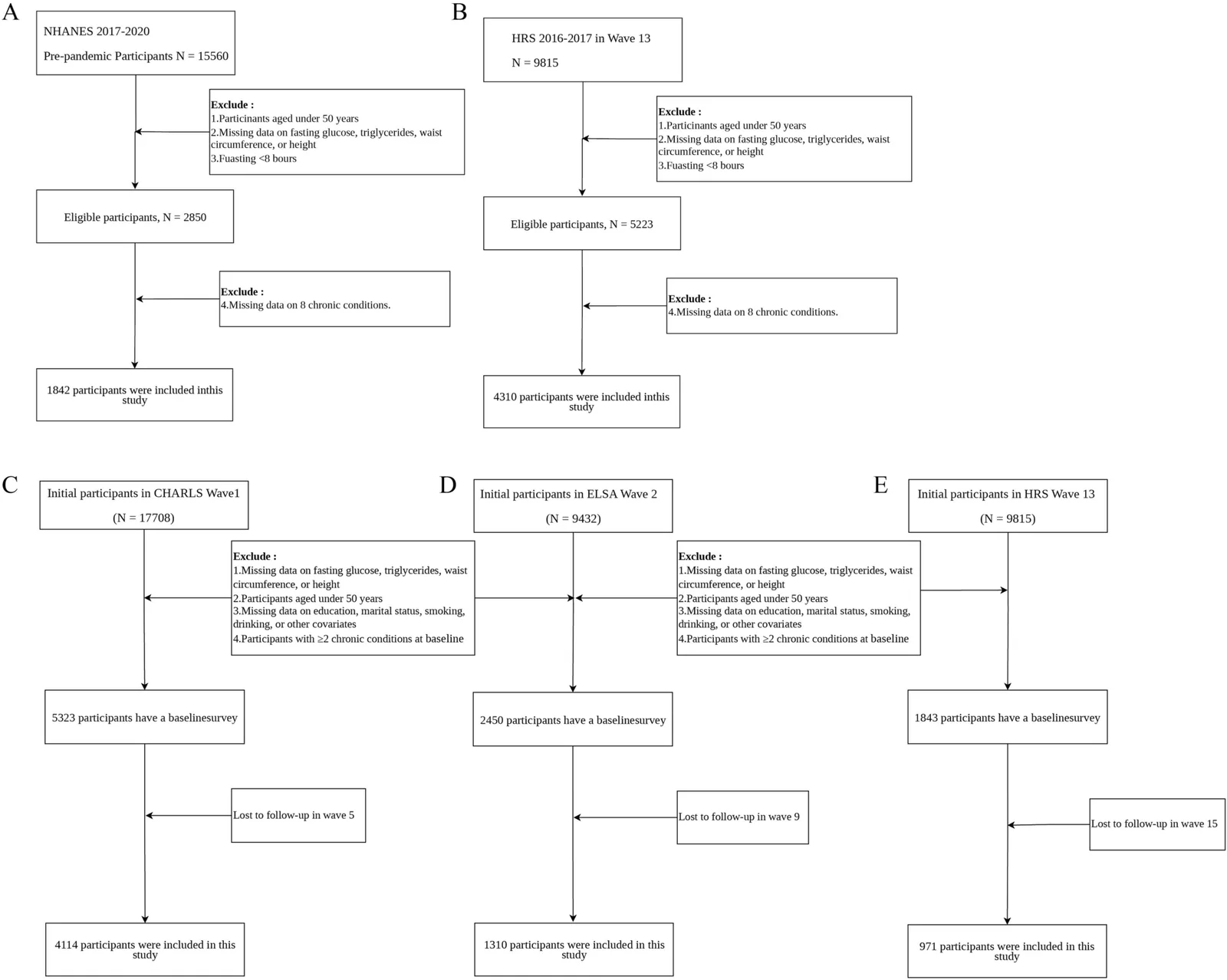
Participant-selection flowcharts for the cross-sectional analyses in NHANES (**A**) and HRS (**B**), and the prospective analyses in CHARLS (**C**), ELSA (**D**), and HRS (**E**). *Abbreviations*: NHANES-National Health and Nutrition Examination Survey, HRS-Health and Retirement Study, CHARLS-China Health and Retirement Longitudinal Study, and ELSA-English Longitudinal Study of Ageing

NHANES is a continuous, nationally representative survey of the non-institutionalized civilian population of the United States, using a complex, multistage probability sampling design. The present study used data from the 2017–March 2020 pre-pandemic cycle. Among 15,560 participants, individuals were excluded if they were aged < 50 years, had a fasting duration of < 8 h, or had missing data on fasting glucose, triglycerides, waist circumference, or height. Of the remaining 2850 eligible participants, those with missing information on any of the eight chronic conditions were further excluded, leaving 1842 participants for the cross-sectional analysis (Fig. 1A). NHANES protocols were approved by the National Center for Health Statistics Research Ethics Review Board, and all participants provided written informed consent.

HRS is a nationally representative biennial longitudinal study of adults aged 50 years or older in the United States. Wave 13, conducted in 2016–2017, was used for the cross-sectional analysis because it included the venous blood and anthropometric measurements required for calculating TyG-related indices. Among 9815 participants, those aged < 50 years, those with a fasting duration of < 8 h, and those with missing fasting glucose, triglycerides, waist circumference, or height were excluded. After further excluding participants with missing information on the eight chronic conditions, 4,310 participants were included in the HRS cross-sectional analysis (Fig. 1B).The 2016 Venous Blood Study respondent weight (PVBSWGTR) was used in HRS weighted analyses to account for differential selection and participation in the venous blood component.

CHARLS is a nationally representative longitudinal study of Chinese adults. Wave 1, conducted in 2011, was used as the baseline, and participants were followed through Wave 5. Of the 17,708 participants enrolled at Wave 1, individuals were excluded if they were aged < 50 years, had missing fasting glucose, triglycerides, waist circumference, or height, had incomplete covariate data, or already had multimorbidity at baseline. A total of 5323 participants were eligible at baseline. After excluding 1209 participants who were lost to follow-up by Wave 5, 4,114 participants were included in the prospective analysis (Fig. 1C). CHARLS was approved by the Biomedical Ethics Review Committee of Peking University (IRB00001052-11015), and written informed consent was obtained from all participants [31].

ELSA is a nationally representative longitudinal study of community-dwelling adults aged 50 years or older in England. Wave 2, conducted in 2004–2005, was used as the baseline because it included the biochemical and anthropometric measurements required for the present study. Participants were followed through Wave 9. Of the 9432 participants at Wave 2, those with missing exposure variables, incomplete covariate data, or baseline multimorbidity were excluded, leaving 2450 eligible participants. After excluding 1140 participants who were lost to follow-up, 1310 participants were included in the prospective analysis (Fig. 1D). ELSA received ethical approval from the London Multicentre Research Ethics Committee, and all participants provided written informed consent [32].

For the prospective HRS analysis, Wave 13 was used as the baseline, and participants were followed through Wave 15. Participants were excluded if they were aged < 50 years, had missing fasting glucose, triglycerides, waist circumference, or height, had incomplete covariate data, or had multimorbidity at baseline. A total of 1843 participants were eligible at baseline. After excluding 872 participants who were lost to follow-up, 971 participants were included in the final prospective analysis (Fig. 1E). HRS protocols were approved by the relevant institutional review board, and all participants provided informed consent.

### Assessment of TyG-related indices

TyG-WHtR was calculated using the following formulas [21, 27]:

$$ TyG = Ln\left[ \begin{gathered} fasting{\mkern 1mu} triglycerides{\mkern 1mu} (mg/dL) \hfill \\ {\mkern 1mu} \times {\mkern 1mu} fasting{\mkern 1mu} glu\cos e{\mkern 1mu} (mg/dL)/2 \hfill \\ \end{gathered} \right] $$

$$\:\mathrm{W}\mathrm{H}\mathrm{t}\mathrm{R}=\frac{\mathrm{w}\mathrm{a}\mathrm{i}\mathrm{s}\mathrm{t}\:\mathrm{c}\mathrm{i}\mathrm{r}\mathrm{c}\mathrm{u}\mathrm{m}\mathrm{f}\mathrm{e}\mathrm{r}\mathrm{e}\mathrm{n}\mathrm{c}\mathrm{e}}{\mathrm{h}\mathrm{e}\mathrm{i}\mathrm{g}\mathrm{h}\mathrm{t}}$$

$$\:\mathrm{T}\mathrm{y}\mathrm{G}-\mathrm{W}\mathrm{H}\mathrm{t}\mathrm{R}=\mathrm{T}\mathrm{y}\mathrm{G}\ast\:\mathrm{W}\mathrm{H}\mathrm{t}\mathrm{R}$$

Fasting triglyceride and glucose concentrations were harmonized to mg/dL across NHANES, CHARLS, ELSA, and HRS before calculation of the TyG index. When originally reported in mmol/L, fasting glucose and triglyceride concentrations were converted to mg/dL by multiplying by 18.018 and 88.57, respectively [33]. 

In addition to TyG-WHtR, five alternative TyG-related indices were evaluated for comparative analyses: TyG-body mass index (TyG-BMI), TyG-waist circumference (TyG-WC), TyG-body roundness index (TyG-BRI), TyG-a body shape index (TyG-ABSI), and TyG-weight-adjusted waist index (TyG-WWI). Detailed formulas, required measurement units, and definitions of these indices are provided in Supplementary Table S1.

### Definition of multimorbidity

The primary outcome was multimorbidity, defined as the coexistence of at least two of eight chronic conditions consistently available across NHANES, CHARLS, ELSA, and HRS: hypertension, dyslipidaemia, diabetes, cancer, chronic lung disease, heart disease, stroke, and arthritis. Both the number and type of conditions included in the primary definition were identical across the four databases, thereby improving the comparability of multimorbidity ascertainment across populations.

The eight chronic conditions were ascertained using harmonized cohort-specific algorithms incorporating self-reported physician diagnoses, condition-specific medication use, and available clinical or laboratory measurements. Hypertension, dyslipidemia, and diabetes were identified if any corresponding diagnostic, treatment, or objective measurement criterion was met, whereas cancer, chronic lung disease, heart disease, stroke, and arthritis were primarily identified from physician-diagnosed conditions.The closest corresponding questionnaire item was selected in each database when the wording of the original questions differed. Heart disease was defined as a composite condition encompassing physician-diagnosed coronary heart disease, myocardial infarction, angina, congestive heart failure, or other physician-diagnosed heart conditions, according to the information available in each cohort. Each condition was coded as present or absent, and the total number of conditions was calculated for each participant. Detailed cohort-specific ascertainment and harmonisation procedures are presented in Supplementary Table S2.

In the cross-sectional analyses of NHANES and HRS, prevalent multimorbidity was defined as the presence of at least two of the eight conditions at the corresponding survey assessment. In the prospective analyses of CHARLS, ELSA, and HRS, participants with fewer than two of the eight conditions at baseline were eligible for inclusion. Incident multimorbidity was defined as the first follow-up wave at which a participant met the criterion of having at least two conditions. Follow-up time was calculated from the baseline interview date to the interview date at which incident multimorbidity was first identified.

As a supplementary analysis, multimorbidity was assessed using a broader, cohort-specific definition based on the presence of at least two of 14 chronic conditions. Because the composition of these 14 conditions differed across cohorts according to questionnaire design and data availability, this broader definition was used only to evaluate the robustness of the primary findings and was not used as the primary harmonised outcome. The cohort-specific composition of the 14-condition definition is presented in Supplementary Table S3.

### Covariates

The prespecified covariate set, selected for clinical relevance and data availability, included age, sex, education, marital status, smoking, drinking, physical activity, household income, health insurance, CRP, and hemoglobin. Age, CRP, and hemoglobin were modeled as continuous variables, whereas sex (male/female), marital status (married/other), and health insurance (insured/uninsured) were dichotomized. Education was harmonized into elementary, high school, and university levels. Due to inter-cohort questionnaire variations, smoking and drinking statuses were aligned using the most comparable category-specific criteria. Physical activity was grouped into low, moderate, and high based on frequency and intensity, while household income was stratified into three tiers using cohort-specific thresholds to account for currency and temporal differences.Race/ethnicity was additionally included in the NHANES models because of the multiethnic sampling design of NHANES. Place of residence was additionally included in the CHARLS models and was categorized as rural or urban. Detailed definitions and harmonization procedures for all covariates are provided in Supplementary Table S4.

### Statistical analyses

#### Cross-sectional analyses in NHANES and HRS

Continuous variables were presented as mean ± standard deviation or median with interquartile range, and categorical variables as numbers and percentages. Group differences were assessed using t test, the Mann–Whitney U test, or the chi-square test, as appropriate. Logistic regression models were fitted separately for NHANES and HRS to estimate odds ratios and 95% confidence intervals for prevalent multimorbidity. TyG-WHtR was analyzed continuously and in cohort-specific quartiles. Model 1 was unadjusted. Model 2 was adjusted for age, sex, education, marital status, health insurance, household income, and physical activity. Model 3 was further adjusted for smoking status, drinking status, CRP, and hemoglobin.Race/ethnicity was additionally adjusted for in NHANES. Restricted cubic splines (RCS) and two-piecewise regression were used to assess nonlinear and threshold associations. Receiver operating characteristic curves (ROC) were used to compare the areas under the curve of TyG-WHtR, TyG-BMI, TyG-WC, TyG-BRI, TyG-ABSI, and TyG-WWI.

#### Prospective analyses in CHARLS, ELSA, and HRS

Cox proportional hazards models were used to estimate hazard ratios (HRs) and 95% CIs for the association between baseline TyG-WHtR and incident multimorbidity in CHARLS, ELSA, and HRS. Participants were followed from baseline until incident multimorbidity, the last completed follow-up, or the end of the study. TyG-WHtR was analyzed continuously and by cohort-specific quartiles using the same three adjustment models, with additional adjustment for rural or urban residence in CHARLS. The proportional hazards assumption was assessed using Schoenfeld residuals. RCS, two-piecewise Cox regression, and likelihood ratio tests were used to examine nonlinear and threshold associations. ROC analyses were performed to compare the discriminatory performance of TyG-WHtR with the other TyG-related indices. Subgroup analyses were performed using multivariable Cox proportional hazards models. Models were adjusted for age, sex, education, marital status, health insurance, physical activity, household income, smoking, drinking, CRP, and hemoglobin, with additional adjustment for residence in CHARLS. The stratifying variable was omitted from the corresponding model. *P* values for interaction were calculated by including a multiplicative interaction term between continuous TyG-WHtR and each subgroup variable.For the longitudinal sensitivity analyses, participants with at least two of the 14 cohort-specific chronic conditions at baseline were excluded, and incident multimorbidity was defined as the first occurrence of at least two of these conditions during follow-up. The Cox regression analyses were repeated using quartile-based TyG-WHtR, with the same adjustment models and follow-up periods as in the primary analyses.

All analyses were performed using R version 4.5.2, with a two-sided * P*value < 0.05 considered statistically significant.

## Results

### Participant selection and baseline characteristics

The cross-sectional analyses included 1842 participants from NHANES and 4,310 from HRS. The longitudinal analyses included 4114 participants from CHARLS, 1310 from ELSA, and 971 from HRS in the analytic datasets. During follow-up, incident multimorbidity occurred in 1714 (41.7%) CHARLS participants, 725 (55.3%) ELSA participants, and 283 (29.1%) HRS participants (Table 1, Table 2 and Supplementary Tables S5–S6).

**Table 1:** Baseline characteristics of participants in the NHANES and HRS cross-sectional analyses

Characteristics	Total	Without multimorbidity	With multimorbidity	*P* value
*NHANES*	**N = 1,842**	**N = 448**	**N = 1,394**	
Age,years	64.37 ± 9.03	61.17 ± 8.31	65.40 ± 9.01	< 0.001
Weight,kg	81.97 ± 20.18	76.61 ± 17.88	83.70 ± 20.58	< 0.001
CRP, mg/L	4.47 ± 8.59	3.14 ± 6.64	4.90 ± 9.09	< 0.001
Hemoglobin, g/dL	14.01 ± 1.55	14.34 ± 1.49	13.90 ± 1.55	< 0.001
*Sex, n(%)*				0.002
Female	942 (51.14)	201 (44.87)	741 (53.16)	
Male	900 (48.86)	247 (55.13)	653 (46.84)	
*Education, n(%)*				0.021
Elementary	398 (21.61)	91 (20.31)	307 (22.02)	
High school	467 (25.35)	94 (20.98)	373 (26.76)	
University	977 (52.99)	264 (58.71)	713 (51.15)	
*Marital status, n(%)*				0.013
Married	1692 (91.86)	424 (94.64)	1268 (90.96)	
Other	150 (8.14)	24 (5.36)	126 (9.04)	
*Smoking status, n(%)*				< 0.001
Current	278 (15.09)	59 (13.17)	219 (15.71)	
Former	576 (31.27)	110 (24.55)	466 (33.43)	
Never	988 (53.64)	279 (62.28)	709 (50.86)	
*Drinking status, n(%)*				0.470
Current	938 (50.92)	239 (53.35)	699 (50.14)	
Former	501 (27.20)	118 (26.34)	383 (27.47)	
Never	403 (21.88)	91 (20.31)	312 (22.38)	
*Race, n(%)*				< 0.001
Mexican American	203 (11.02)	58 (12.95)	145 (10.40)	
Non-Hispanic Black	478 (25.95)	103 (22.99)	375 (26.90)	
Non-Hispanic White	714 (38.76)	146 (32.59)	568 (40.75)	
Other	228 (12.38)	93 (20.76)	135 (9.68)	
Other Hispanic	219 (11.89)	48 (10.71)	171 (12.27)	
*Insurance, n(%)*				< 0.001
No	176 (9.55)	63 (14.06)	113 (8.11)	
Yes	1666 (90.45)	385 (85.94)	1281 (91.89)	
*Income group, n(%)*				< 0.001
Low	614 (33.33)	118 (26.34)	496 (35.58)	
Medium	614 (33.33)	145 (32.37)	469 (33.64)	
High	614 (33.33)	185 (41.29)	429 (30.77)	
*Physical group, n(%)*				0.010
Low	614 (33.33)	131 (29.24)	483 (34.65)	
Medium	614 (33.33)	175 (39.06)	439 (31.49)	
High	614 (33.33)	142 (31.70)	472 (33.86)	
*HRS*	**N = 4,310**	**N = 1,133**	**N = 3,177**	
Age,years	68.87 ± 9.87	64.31 ± 8.75	70.49 ± 9.74	< 0.001
Weight,kg	82.18 ± 18.33	78.85 ± 17.27	83.39 ± 18.56	< 0.001
Hemoglobin, g/dL	13.53 ± 1.54	13.87 ± 1.40	13.41 ± 1.57	< 0.001
CRP, mg/L	4.76 ± 10.79	3.78 ± 6.89	5.11 ± 11.86	< 0.001
*Sex, n(%)*				0.013
Female	2498 (57.96)	692 (61.08)	1806 (56.85)	
Male	1812 (42.04)	441 (38.92)	1371 (43.15)	
*Education, n(%)*				< 0.001
Elementary school	741 (17.19)	151 (13.33)	590 (18.57)	
High school	2550 (59.16)	643 (56.75)	1907 (60.03)	
University	1019 (23.64)	339(29.92)	680 (21.40)	
*Marital status, n(%)*				< 0.001
Married	2569 (59.61)	748 (66.02)	1821 (57.32)	
Others	1741 (40.39)	385 (33.98)	1356 (42.68)	
*Insurance, n(%)*				< 0.001
No	1595 (37.01)	688(60.72)	907 (28.55)	
Yes	2715 (62.99)	445 (39.28)	2270 (71.45)	
*Smoking status, n(%)*				0.218
No	3819 (88.61)	992(87.56)	2827(88.98)	
Yes	491(11.39)	141 (12.44)	350 (11.02)	
*Drinking status, n(%)*				< 0.001
No	1839 (42.67)	383 (33.80)	1456 (45.83)	
Yes	2471 (57.33)	750 (66.20)	1721 (54.17)	
*Physical group, n(%)*				< 0.001
Low	887 (20.58)	126 (11.12)	761(23.95)	
Medium	1308 (30.35)	327 (28.86)	981 (30.88)	
High	2115 (49.07)	680 (60.02)	1435 (45.17)	
*Income group, n(%)*				< 0.001
Low	1437 (33.34)	237 (20.92)	1200 (37.77)	
Medium	1436 (33.32)	356 (31.42)	1080 (33.99)	
High	1437 (33.34)	540 (47.66)	897 (28.23)	

Data are presented as mean ± SD for continuous variables and n (%) for categorical variables.*P* values were calculated using independent-samples t tests, Mann–Whitney U tests, or Pearson chi-square tests, as appropriate.Boldface indicates column headings and cohort-specific sample-size entries and does not denote statistical significance.

Abbreviations: CRP, C-reactive protein; Hb, hemoglobin; HRS, Health and Retirement Study; NHANES, National Health and Nutrition Examination Survey; SD, standard deviation

**Table 2:** Baseline characteristics of participants in CHARLS according to incident multimorbidity status

Characteristic	Overall (n = 4114)	No incident multimorbidity (n = 2400)	Incident multimorbidity (n = 1714)	*P* value
Age, years	60.71 ± 7.33	60.44 ± 7.47	61.08 ± 7.12	0.005
Weight, kg	57.17 ± 10.72	55.84 ± 10.10	59.03 ± 11.26	< 0.001
CRP,mg/L	2.42 ± 6.90	2.36 ± 7.61	2.52 ± 5.77	0.448
Hemoglobin, g/dL	14.35 ± 2.17	14.27 ± 2.14	14.47 ± 2.20	0.003
*Sex, n (%)*				**0.014**
Female	2122 (51.58)	1199 (49.96)	923 (53.85)	
Male	1992 (48.42)	1201 (50.04)	791 (46.15)	
*Education, n (%)*				**0.831**
Elementary school	3769 (91.61)	2204 (91.83)	1565 (91.31)	
High school	319 (7.75)	181 (7.54)	138 (8.05)	
University	26 (0.63)	15 (0.62)	11 (0.64)	
*Residence, n (%)*				**0.075**
Rural	2868 (69.71)	1699 (70.79)	1169 (68.20)	
Urban	1246 (30.29)	701 (29.21)	545 (31.80)	
*Marital status, n (%)*				**0.026**
Married	3633 (88.31)	2142 (89.25)	1491 (86.99)	
Other	481 (11.69)	258 (10.75)	223 (13.01)	
*Health insurance, n (%)*				**0.030**
No	209 (5.08)	137 (5.71)	72 (4.20)	
Yes	3905 (94.92)	2263 (94.29)	1642 (95.80)	
*Smoking status, n (%)*				**0.023**
No	2751 (66.87)	1571 (65.46)	1180 (68.84)	
Yes	1363 (33.13)	829 (34.54)	534 (31.16)	
*Drinking status, n (%)*				**0.039**
No	2664 (64.75)	1523 (63.46)	1141 (66.57)	
Yes	1450 (35.25)	877 (36.54)	573 (33.43)	
*Physical activity, n (%)*				**0.929**
High (5–7 days/week)	1819 (44.21)	1067 (44.46)	752 (43.87)	
Low (0–2 days/week)	1627 (39.55)	946 (39.42)	681 (39.73)	
Moderate (3–4 days/week)	668 (16.24)	387 (16.12)	281 (16.39)	
*Household income, n (%)*				**0.107**
High	1371 (33.33)	827 (34.46)	544 (31.74)	
Low	1372 (33.35)	773 (32.21)	599 (34.95)	
Middle	1371 (33.33)	800 (33.33)	571 (33.31)	

Data are presented as mean ± SD or median (interquartile range) for continuous variables, as appropriate, and n (%) for categorical variables. *P *values were calculated using the independent-samples t test, Mann–Whitney U test, or chi-square test, as appropriate

In the cross-sectional samples, prevalent multimorbidity was present in 1394 (75.7%) NHANES participants and 3,177 (73.7%) HRS participants. In both cohorts, participants with multimorbidity were older, had greater body weight and higher CRP concentrations, and had lower hemoglobin concentrations than those without multimorbidity (all *P* < 0.001). Multimorbidity status also differed by sex, education, marital status, health insurance, and physical activity in both cohorts. Additional differences were observed for smoking, race/ethnicity, and income in NHANES and for drinking status in HRS (Table 1).

Across the prospective cohorts, participants who developed multimorbidity were older and had greater body weight than those who remained free of multimorbidity. Baseline profiles additionally differed by sex, marital status, health insurance, smoking, and drinking in CHARLS; by education and income in ELSA; and by sex, education, health insurance, smoking, drinking, and physical activity in HRS (Table 2 and Supplementary Tables S5–S6).

### Cross-sectional associations of TyG-WHtR with prevalent multimorbidity

TyG-WHtR was positively associated with prevalent multimorbidity in both cross-sectional cohorts (Table 3). In the fully adjusted model, each 1-unit increase in TyG-WHtR was associated with higher odds of multimorbidity in NHANES (OR 2.16, 95% CI 1.75–2.67; *P* < 0.001) and HRS (OR 1.64, 95% CI 1.46–1.83; *P* < 0.001). Compared with Q1, the fully adjusted ORs for Q4 were 5.04 (95% CI 3.02–8.42; *P* < 0.001) in NHANES and 3.63 (95% CI 2.70–4.90; *P* < 0.001) in HRS, with progressively larger estimates across quartiles in both cohorts.

**Table 3:** Cross-sectional associations of continuous and quartile-based TyG-WHtR with prevalent multimorbidity in NHANES and HRS

Cohort	TyG-WHtR exposure	Model 1, OR (95% CI); *P* value	Model 2, OR (95% CI); *P* value	Model 3, OR (95% CI); *P* value
NHANES	Continuous (per 1-unit increase)	2.07 (1.82–2.36); *P* < 0.001	2.11 (1.72–2.58); *P* < 0.001	2.16 (1.75–2.67); *P* < 0.001
	Q1	1.00 (Reference)	1.00 (Reference)	1.00 (Reference)
	Q2	1.56 (1.18–2.05); *P* = 0.002	1.50 (1.10–2.04); *P* = 0.011	1.61 (1.18–2.21); *P* = 0.003
	Q3	2.56 (1.91–3.44); *P* < 0.001	2.23 (1.55–3.21); *P* < 0.001	2.34 (1.61–3.39); *P* < 0.001
	Q4	6.39 (4.42–9.24); *P* < 0.001	5.04 (3.03–8.38); *P* < 0.001	5.04 (3.02–8.42); *P* < 0.001
HRS	Continuous (per 1-unit increase)	1.71 (1.58–1.84); *P* < 0.001	1.62 (1.45–1.81); *P* < 0.001	1.64 (1.46–1.83); *P* < 0.001
	Q1	1.00 (Reference)	1.00 (Reference)	1.00 (Reference)
	Q2	1.71 (1.43–2.05); *P* < 0.001	1.53 (1.24–1.88); *P* < 0.001	1.50 (1.21–1.85); *P* < 0.001
	Q3	2.34 (1.94–2.83); *P* < 0.001	1.99 (1.57–2.52); *P* < 0.001	2.11 (1.65–2.68); *P* < 0.001
	Q4	4.23 (3.42–5.23); *P* < 0.001	3.60 (2.69–4.83); *P* < 0.001	3.63 (2.70–4.90); *P* < 0.001

Values are odds ratios (ORs) with 95% confidence intervals (CIs) and* P* values. For the continuous analysis, the OR represents a 1-unit increase in TyG-WHtR. Q1 is the reference category in the quartile analysis

Model 1 was unadjusted. In NHANES, Model 2 was adjusted for sex, education, marital status, household income, race/ethnicity,health insurance, physical activity and age; Model 3 was additionally adjusted for smoking, drinking, CRP, and hemoglobin. In HRS, Model 2 was adjusted for sex, education, marital status, household income,insurance, physical activity group and age; Model 3 was additionally adjusted for smoking, drinking, hemoglobin, and CRP

*Abbreviations*: CI, confidence interval; OR, odds ratio; Q, quartile; TyG-WHtR, triglyceride-glucose waist-to-height ratio

**Fig. 2:**
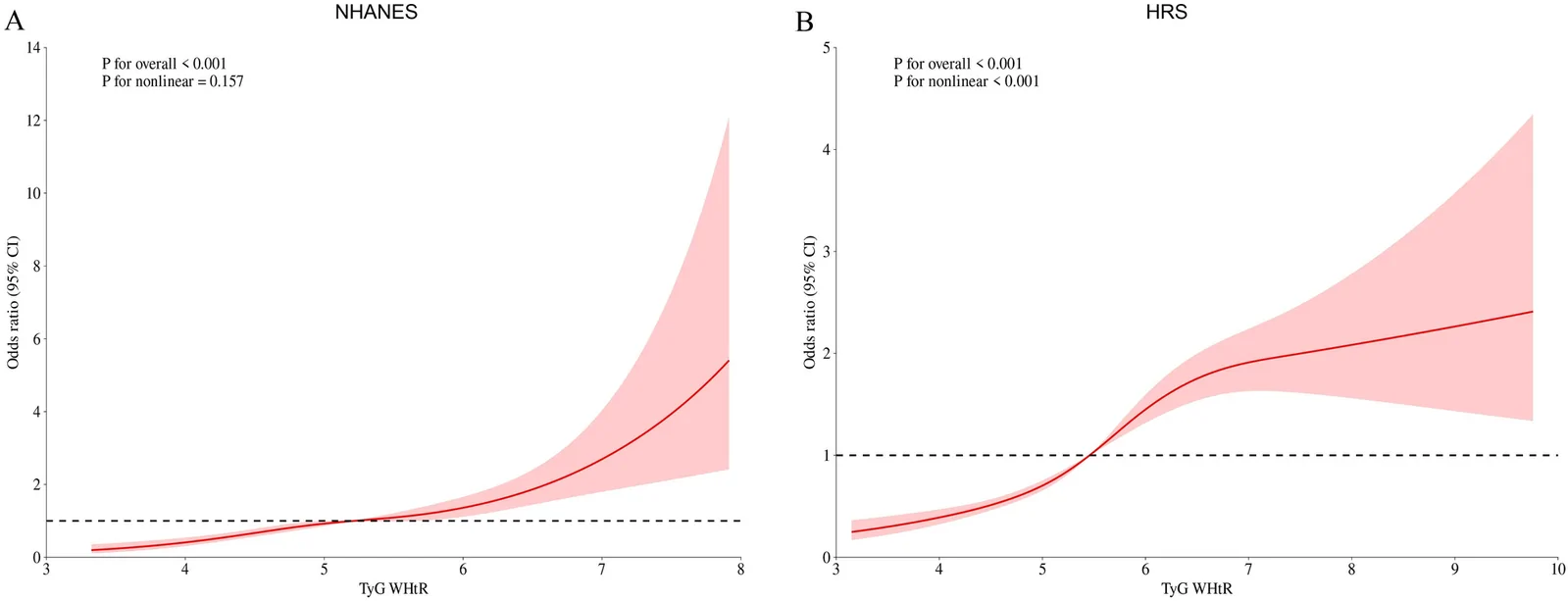
Restricted cubic spline associations of TyG-WHtR with prevalent multimorbidity in (**A**) NHANES and **B** HRS. Solid lines represent odds ratios and shaded areas represent 95% confidence intervals

Restricted cubic spline analyses supported an overall association in both cohorts (both P for overall < 0.001; Fig. 2). The association was approximately linear in NHANES (*P* for nonlinearity = 0.157), whereas a significant nonlinear pattern was observed in HRS (*P* for nonlinearity < 0.001).

**Fig. 3:**
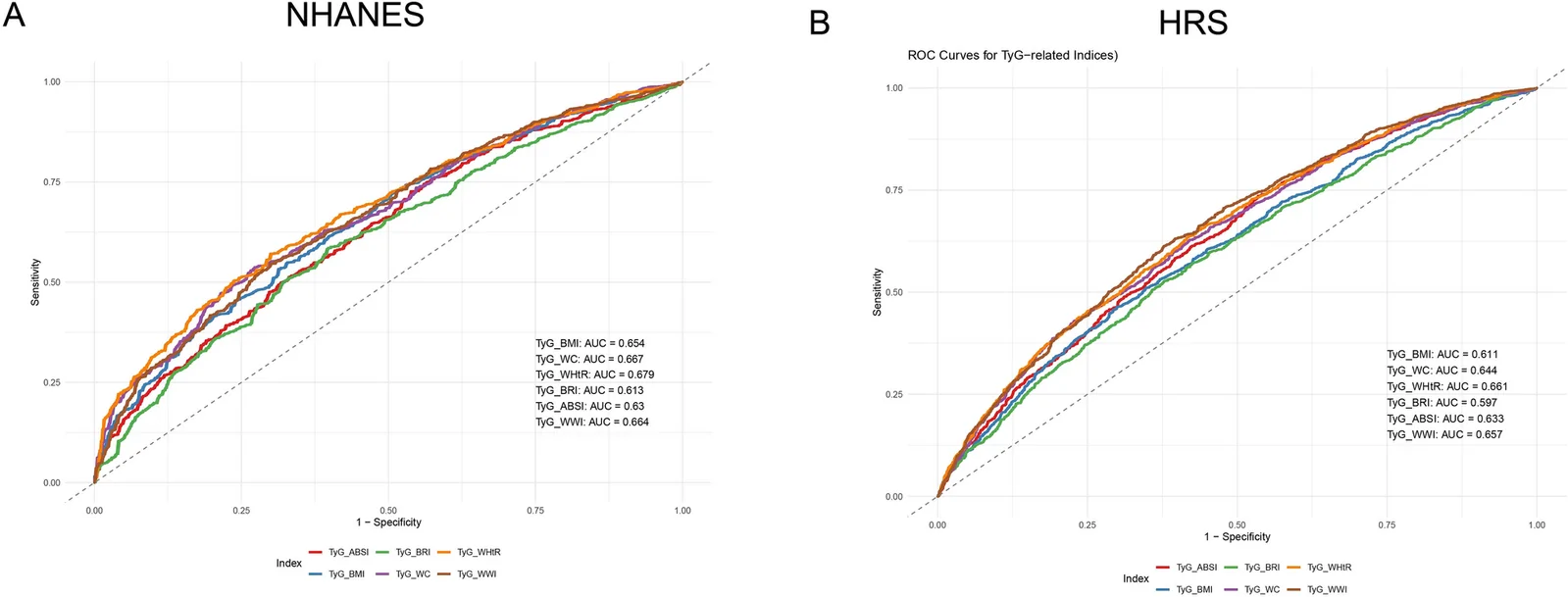
Receiver operating characteristic curves comparing TyG-related anthropometric indices for prevalent multimorbidity in (**A**) NHANES and **B** HRS

The discriminatory ability of all TyG-related anthropometric indices was modest (Fig. 3). TyG-WHtR yielded the largest AUC in NHANES (0.679), followed by TyG-WC (0.667), and also yielded the largest AUC in HRS (0.661), narrowly exceeding TyG-WWI (0.657).

### Longitudinal associations of TyG-WHtR with incident multimorbidity

In the prospective analyses, higher TyG-WHtR was associated with incident multimorbidity, although the strength of association varied across cohorts (Table 4). In fully adjusted continuous models, each 1-unit increase in TyG-WHtR was associated with a 15% higher risk in CHARLS (HR 1.15, 95% CI 1.10–1.19; *P* < 0.001) and a 17% higher risk in ELSA (HR 1.17, 95% CI 1.08–1.26; *P* < 0.001), whereas the corresponding estimate in HRS was not statistically significant (HR 1.06, 95% CI 0.96–1.16; *P* = 0.264). In quartile analyses, however, Q4 was associated with higher risk than Q1 in all three cohorts: HR 1.87 (95% CI 1.59–2.21; *P* < 0.001) in CHARLS, 1.67 (95% CI 1.30–2.15; *P* < 0.001) in ELSA, and 1.57 (95% CI 1.08–2.29; *P* = 0.018) in HRS. Associations for intermediate quartiles were evident in CHARLS and ELSA but were less consistent in HRS.

**Table 4:** Associations of continuous and quartile-based TyG-WHtR with incident multimorbidity in CHARLS, ELSA, and HRS

Cohort	TyG-WHtR	Model 1		Model 2		Model 3	
		HR (95% CI)	*P* value	HR (95% CI)	*P* value	HR (95% CI)	*P* value
CHARLS	Continuous	1.19 (1.15–1.23)	< 0.001	1.15 (1.11–1.19)	< 0.001	1.15 (1.10–1.19)	< 0.001
	Q1	1.00 (reference)	–	1.00 (reference)	–	1.00 (reference)	–
	Q2	1.11 (0.96–1.29)	0.168	1.08 (0.93–1.26)	0.303	1.08 (0.93–1.26)	0.320
	Q3	1.40 (1.21–1.62)	< 0.001	1.32 (1.14–1.54)	< 0.001	1.32 (1.13–1.54)	< 0.001
	Q4	2.12 (1.85–2.42)	< 0.001	1.88 (1.60–2.22)	< 0.001	1.87 (1.59–2.21)	< 0.001
ELSA	Continuous	1.15 (1.08–1.23)	< 0.001	1.15 (1.06–1.23)	< 0.001	1.17 (1.08–1.26)	< 0.001
	Q1	1.00 (reference)	–	1.00 (reference)	–	1.00 (reference)	–
	Q2	1.30 (1.04–1.64)	0.023	1.25 (0.99–1.59)	0.057	1.25 (0.98–1.58)	0.068
	Q3	1.74 (1.39–2.17)	< 0.001	1.67 (1.32–2.12)	< 0.001	1.67 (1.31–2.11)	< 0.001
	Q4	1.71 (1.38–2.14)	< 0.001	1.64 (1.29–2.09)	< 0.001	1.67 (1.30–2.15)	< 0.001
HRS	Continuous	1.09 (1.01–1.16)	0.017	1.06 (0.97–1.16)	0.232	1.06 (0.96–1.16)	0.264
	Q1	1.00 (reference)	–	1.00 (reference)	–	1.00 (reference)	–
	Q2	1.18 (0.82–1.71)	0.375	1.11 (0.76–1.62)	0.583	1.22 (0.82–1.80)	0.323
	Q3	1.43 (1.01–2.04)	0.045	1.37 (0.95–1.97)	0.093	1.36 (0.93–2.00)	0.115
	Q4	1.66 (1.17–2.34)	0.004	1.57 (1.10–2.23)	0.013	1.57 (1.08–2.29)	0.018

Data are presented as HR (95% CI). Continuous estimates are reported per 1-unit increase in TyG-WHtR. Model 1 was unadjusted. Model 2 was adjusted for sex, education, marital status, health insurance, physical activity, household income, and age.Residence was additionally included in CHARLS. Model 3 was further adjusted for smoking, drinking, CRP, and hemoglobin

*Abbreviations*: CHARLS, China Health and Retirement Longitudinal Study; CI, confidence interval; CRP, C-reactive protein; ELSA, English Longitudinal Study of Ageing; HR, hazard ratio; HRS, Health and Retirement Study; TyG-WHtR, triglyceride-glucose waist-to-height ratio index

### Nonlinear and threshold associations in the prospective cohorts

**Fig. 4:**
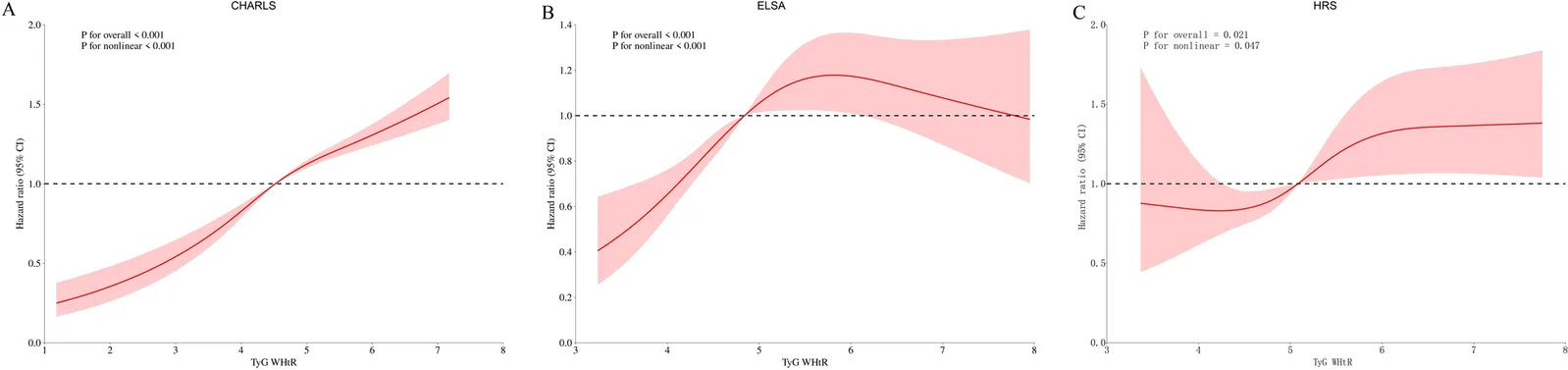
Nonlinear and threshold associations of TyG-WHtR with incident multimorbidity. Restricted cubic spline curves are shown for (**A**) CHARLS, **B** ELSA, and **C** HRS. Solid lines represent hazard ratios and shaded areas represent 95% confidence intervals

Restricted cubic spline analyses indicated nonlinear associations between TyG-WHtR and incident multimorbidity in CHARLS (*P* for overall < 0.001;*P* for nonlinearity < 0.001), ELSA (*P* for overall < 0.001;*P* for nonlinearity < 0.001), and HRS (*P* for overall = 0.021;*P* for nonlinearity = 0.047) (Fig. 4A-C). Segmented regression identified inflection points of 5.92, 5.80, and 6.69, respectively (Table 5). Below these inflection points, each 1-unit increase in TyG-WHtR was associated with higher multimorbidity risk in CHARLS (HR 1.42, 95% CI 1.32–1.54; *P* < 0.001), ELSA (HR 1.98, 95% CI 1.55–2.54; *P* < 0.001), and HRS (HR 1.19, 95% CI 1.00–1.42; *P* = 0.048). Above the inflection points, none of the associations was statistically significant. Likelihood-ratio tests favored the two-piecewise models in all three cohorts (*P* < 0.001, *P* = 0.029, and *P* = 0.022, respectively).

**Table 5:** Threshold analyses of the association between TyG-WHtR and incident multimorbidity in CHARLS, ELSA, and HRS

Cohort	Inflection point	Below inflection point, HR (95% CI)	*P* value	At/above inflection point, HR (95% CI)	*P* value	P for likelihood-ratio test
CHARLS	5.92	1.42 (1.32–1.54)	< 0.001	0.97 (0.85–1.10)	0.614	< 0.001
ELSA	5.80	1.98 (1.55–2.54)	< 0.001	1.26 (0.80–1.99)	0.317	0.029
HRS	6.69	1.19 (1.00–1.42)	0.048	0.85 (0.69–1.05)	0.137	0.022

Threshold analyses were performed using segmented regression. The likelihood-ratio test compared the standard linear model with the two-piecewise model. Effect estimates are presented as HR (95% CI). *Abbreviations*: CHARLS, China Health and Retirement Longitudinal Study; CI, confidence interval; ELSA, English Longitudinal Study of Ageing; HR, hazard ratio; HRS, Health and Retirement Study; TyG-WHtR, triglyceride-glucose waist-to-height ratio index

### Discriminatory performance in the prospective cohorts

**Fig. 5:**
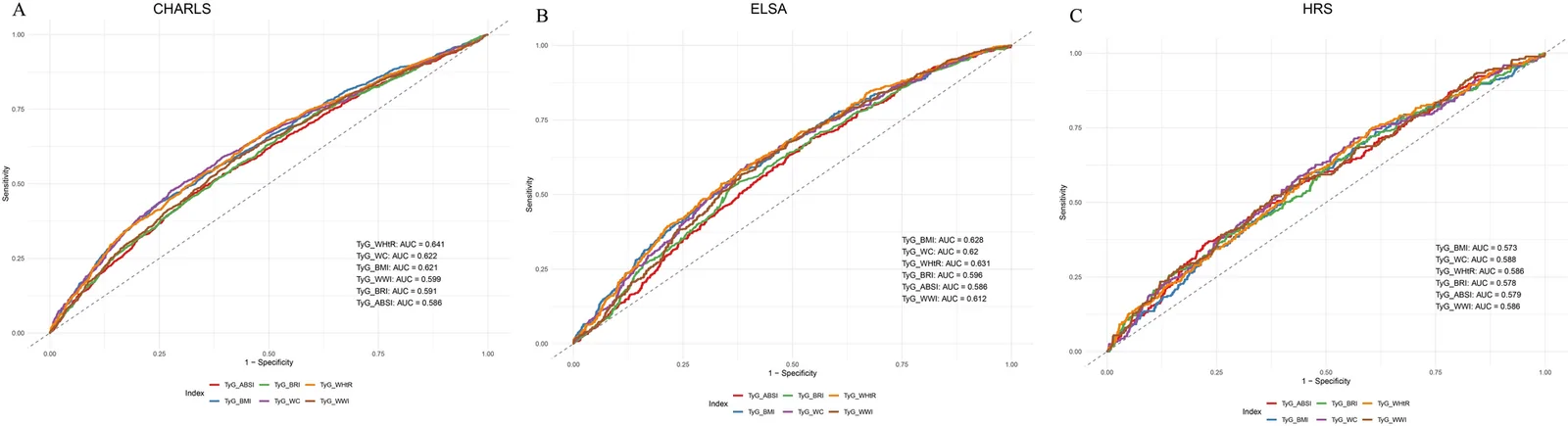
Receiver operating characteristic curves comparing TyG-related anthropometric indices for incident multimorbidity in (**A**) CHARLS, **B** ELSA, and **C** HRS

ROC analyses again showed modest discrimination (Fig. 5). TyG-WHtR had the largest AUC in CHARLS (0.641) and ELSA (0.631). In HRS, TyG-WC had the largest AUC (0.588), while the AUC for TyG-WHtR was 0.586, equal to that of TyG-WWI. Thus, the relative performance of TyG-WHtR was strongest in CHARLS and ELSA but was not superior to all alternative indices in HRS.

### Subgroup analyses

**Fig. 6:**
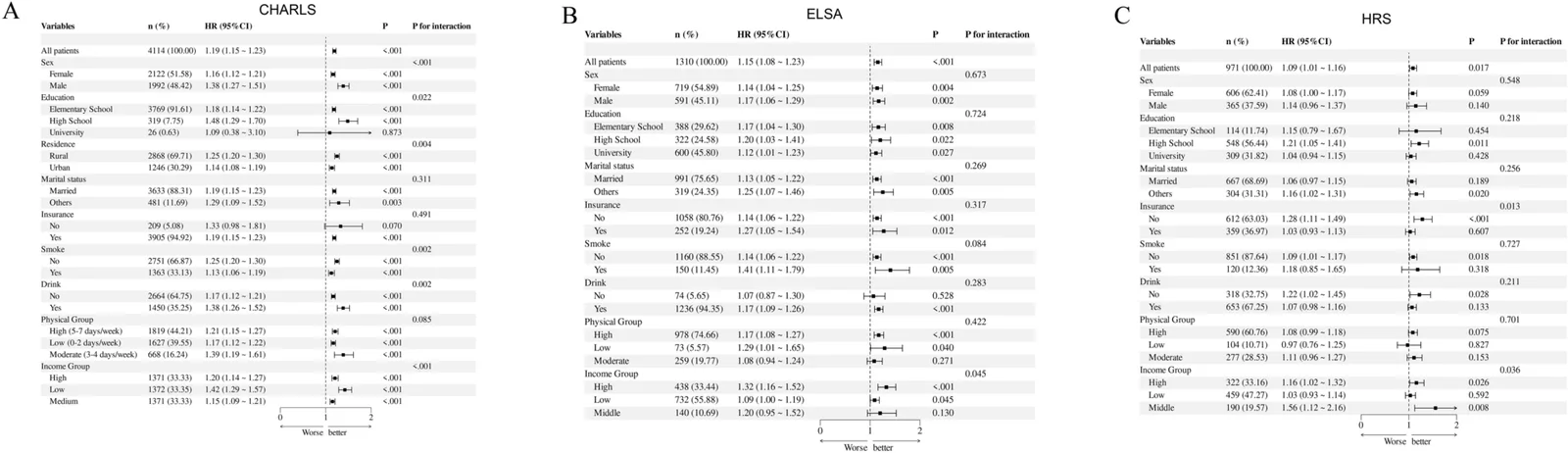
Subgroup analyses of the association between TyG-WHtR and incident multimorbidity in (**A**) CHARLS, **B** ELSA, and **C** HRS

Subgroup analyses are shown in Fig. 6 and Table 6. In CHARLS, the association between TyG-WHtR and incident multimorbidity differed by sex (*P* for interaction < 0.001), education (*P* = 0.022), residence (*P* = 0.004), smoking (*P* = 0.002), drinking (*P* = 0.002), and income (*P* < 0.001). In ELSA, evidence of effect modification was observed only for income (*P* for interaction = 0.045). In HRS, interactions were detected for health insurance (*P* = 0.013) and income (*P* = 0.036). No other tested interactions reached statistical significance.

**Table 6:** Subgroup analyses of the association between TyG-WHtR and incident multimorbidity in CHARLS, ELSA, and HRS

Subgroup	Category	n (%)	HR (95% CI)	*P* value	P for interaction
*CHARLS*					
Overall	All participants	4114 (100.00)	1.19 (1.15–1.23)	< 0.001	–
Sex	Female	2122 (51.58)	1.16 (1.12–1.21)	< 0.001	< 0.001
	Male	1992 (48.42)	1.38 (1.27–1.51)	< 0.001	
Education	Elementary school	3769 (91.61)	1.18 (1.14–1.22)	< 0.001	0.022
	High school	319 (7.75)	1.48 (1.29–1.70)	< 0.001	
	University	26 (0.63)	1.09 (0.38–3.10)	0.873	
Residence	Rural	2868 (69.71)	1.25 (1.20–1.30)	< 0.001	0.004
	Urban	1246 (30.29)	1.14 (1.08–1.19)	< 0.001	
Marital status	Married	3633 (88.31)	1.19 (1.15–1.23)	< 0.001	0.311
	Other	481 (11.69)	1.29 (1.09–1.52)	0.003	
Health insurance	No	209 (5.08)	1.33 (0.98–1.81)	0.070	0.491
	Yes	3905 (94.92)	1.19 (1.15–1.23)	< 0.001	
Smoking	No	2751 (66.87)	1.25 (1.20–1.30)	< 0.001	0.002
	Yes	1363 (33.13)	1.13 (1.06–1.19)	< 0.001	
Drinking	No	2664 (64.75)	1.17 (1.12–1.21)	< 0.001	0.002
	Yes	1450 (35.25)	1.38 (1.26–1.52)	< 0.001	
Physical activity	High (5–7 days/week)	1819 (44.21)	1.21 (1.15–1.27)	< 0.001	0.085
	Low (0–2 days/week)	1627 (39.55)	1.17 (1.12–1.22)	< 0.001	
	Moderate (3–4 days/week)	668 (16.24)	1.39 (1.19–1.61)	< 0.001	
Income	High	1371 (33.33)	1.20 (1.14–1.27)	< 0.001	< 0.001
	Low	1372 (33.35)	1.42 (1.29–1.57)	< 0.001	
	Middle	1371 (33.33)	1.15 (1.09–1.21)	< 0.001	
*ELSA*					
Overall	All participants	1310 (100.00)	1.15 (1.08–1.23)	< 0.001	–
Sex	Female	719 (54.89)	1.14 (1.04–1.25)	0.004	0.673
	Male	591 (45.11)	1.17 (1.06–1.29)	0.002	
Education	Elementary school	388 (29.62)	1.17 (1.04–1.30)	0.008	0.724
	High school	322 (24.58)	1.20 (1.03–1.41)	0.022	
	University	600 (45.80)	1.12 (1.01–1.23)	0.027	
Marital status	Married	991 (75.65)	1.13 (1.05–1.22)	< 0.001	0.269
	Other	319 (24.35)	1.25 (1.07–1.46)	0.005	
Health insurance	No	1058 (80.76)	1.14 (1.06–1.22)	< 0.001	0.317
	Yes	252 (19.24)	1.27 (1.05–1.54)	0.012	
Smoking	No	1160 (88.55)	1.14 (1.06–1.22)	< 0.001	0.084
	Yes	150 (11.45)	1.41 (1.11–1.79)	0.005	
Drinking	No	74 (5.65)	1.07 (0.87–1.30)	0.528	0.283
	Yes	1236 (94.35)	1.17 (1.09–1.26)	< 0.001	
Physical activity	High	978 (74.66)	1.17 (1.08–1.27)	< 0.001	0.422
	Low	73 (5.57)	1.29 (1.01–1.65)	0.040	
	Moderate	259 (19.77)	1.08 (0.94–1.24)	0.271	
Income	High	438 (33.44)	1.32 (1.16–1.52)	< 0.001	0.045
	Low	732 (55.88)	1.09 (1.00–1.19)	0.045	
	Middle	140 (10.69)	1.20 (0.95–1.52)	0.130	
*HRS*					
Overall	All participants	971 (100.00)	1.09 (1.01–1.16)	0.017	–
Sex	Female	606 (62.41)	1.08 (1.00–1.17)	0.059	0.548
	Male	365 (37.59)	1.14 (0.96–1.37)	0.140	
Education	Elementary school	114 (11.74)	1.15 (0.79–1.67)	0.454	0.218
	High school	548 (56.44)	1.21 (1.05–1.41)	0.011	
	University	309 (31.72)	1.04 (0.94–1.15)	0.427	
Marital status	Married	667 (68.69)	1.06 (0.97–1.15)	0.189	0.256
	Other	304 (31.31)	1.16 (1.02–1.31)	0.020	
Health insurance	No	612 (63.03)	1.28 (1.11–1.49)	0.001	0.013
	Yes	359 (36.97)	1.03 (0.93–1.13)	0.607	
Smoking	No	851 (87.64)	1.09 (1.01–1.17)	0.018	0.727
	Yes	120(12.36)	1.16 (0.82–1.62)	0.401	
Drinking	No	318 (32.75)	1.22 (1.02–1.45)	0.028	0.211
	Yes	653 (67.25)	1.07 (0.98–1.16)	0.133	
Physical activity	High	590 (60.76)	1.08 (0.99–1.18)	0.075	0.701
	Low	104 (10.71)	0.97 (0.76–1.25)	0.827	
	Moderate	277 (28.53)	1.11 (0.96–1.28)	0.152	
Income	High	322 (33.16)	1.16 (1.02–1.32)	0.026	0.036
	Low	459 (47.27)	1.03 (0.93–1.14)	0.592	
	Middle	190 (19.57)	1.56 (1.12–2.16)	0.008	

Subgroup analyses were performed using multivariable Cox proportional hazards models. Models were adjusted for age, sex, education, marital status, health insurance, physical activity, household income, smoking, drinking, CRP, and hemoglobin, with additional adjustment for residence in CHARLS. The stratifying variable was omitted from the corresponding model.* P* values for interaction were calculated by including a multiplicative interaction term between continuous TyG-WHtR and each subgroup variable

### Sensitivity analyses using alternative multimorbidity definitions and lagged follow-up

The sensitivity analyses yielded results consistent with the primary findings. Compared with Q1, the adjusted HRs for Q4 in CHARLS were 1.65 (95% CI 1.43–1.90) after excluding events occurring within the first two years, 2.38 (95% CI 1.87–3.04) when multimorbidity was defined as at least three of the eight conditions, and 1.80 (95% CI 1.59–2.04) using the 14-condition definition. The corresponding HRs in ELSA were 1.59 (95% CI 1.18–2.13), 1.83 (95% CI 1.32–2.53), and 1.33 (95% CI 1.09–1.61), respectively. All analyses showed significant trends across quartiles (*P* for trend < 0.001; Table 7 and Supplementary Table S7). The RCS analyses showed dose–response patterns broadly consistent with the primary findings. Participant selection for the 14-condition analyses is shown in Supplementary Fig. S1, and the corresponding RCS curves are presented in Supplementary Figs. S2 and S3.

**Table 7:** Sensitivity analyses of the association between TyG-WHtR quartiles and incident multimorbidity in CHARLS

Analysis	N	Q2 vs Q1, HR (95% CI)	Q3 vs Q1, HR (95% CI)	Q4 vs Q1, HR (95% CI)	*P* for trend
Primary analysis: ≥ 2 of 8 harmonised conditions	4,114	1.08 (0.93–1.26)	1.32 (1.13–1.54)	1.87 (1.59–2.21)	< 0.001
Excluding incident events within the first 2 years	2,513	1.19 (0.98–1.44)	1.37 (1.02–1.58)	1.65 (1.43–1.90)	< 0.001
Stricter definition: ≥ 3 of 8 harmonised conditions	4,114	0.99 (0.77–1.26)	1.47 (1.16–1.85)	2.38 (1.87–3.04)	< 0.001
Expanded definition: ≥ 2 of 14 CHARLS-specific conditions	4,478	1.12 (0.98–1.27)	1.31 (1.15–1.49)	1.80 (1.59–2.04)	< 0.001

Values are hazard ratios (HRs) with 95% confidence intervals (CIs). Q1 was the reference category. Model 3 was adjusted for age, sex, education, marital status, health insurance, physical activity, household income, place of residence, smoking, drinking, C-reactive protein, and hemoglobin. The primary, lagged, and stricter-definition analyses used the eight harmonised disease domains; the expanded analysis used 14 CHARLS-specific chronic conditions.* Abbreviations*: CHARLS, China Health and Retirement Longitudinal Study; CI, confidence interval; HR, hazard ratio; Q, quartile; TyG-WHtR, triglyceride-glucose waist-to-height ratio

## Discussion

This multicohort study provides cross-sectional and prospective evidence that a higher triglyceride-glucose waist-to-height ratio (TyG-WHtR) is associated with a greater burden of multimorbidity among middle-aged and older adults in China, England, and the United States. Four findings are particularly noteworthy. First, the association with prevalent multimorbidity was strong and graded in both NHANES and HRS, with fully adjusted odds ratios of 5.04 and 3.63, respectively, for the highest versus lowest quartile. Second, the prospective analyses supported temporal consistency: the highest quartile was associated with incident multimorbidity in CHARLS, ELSA, and HRS, although the continuous association was attenuated after adjustment in the smaller HRS sample. Third, TyG-WHtR generally showed the strongest or near-strongest discrimination among the six TyG-related anthropometric indices, particularly in the two cross-sectional samples and in CHARLS and ELSA; however, its advantage was small and not uniform in prospective HRS. Fourth, the longitudinal dose–response associations were nonlinear in all three cohorts. Segmented models identified cohort-specific inflection points at 5.92 in CHARLS, 5.80 in ELSA, and 6.69 in HRS, with risk increasing mainly below these values and no clear additional increase above them. Results remained directionally consistent after excluding early events and applying stricter or broader multimorbidity definitions.

These findings extend a literature that has mainly considered individual cardiometabolic outcomes or cardiometabolic multimorbidity. In CHARLS, Lv et al. reported a positive association between TyG-WHtR and incident cardiometabolic multimorbidity defined by diabetes, heart disease, and stroke [21]. Other Chinese studies have similarly linked TyG-derived adiposity indices with cardiometabolic multimorbidity [29, 30], while a recent Shanghai cohort showed that TyG was associated with transitions from a cardiovascular disease-free state to a first cardiovascular event and subsequently to cardiovascular multimorbidity [34]. Evidence from the Tehran Lipid and Glucose Study further demonstrated associations of TyG and its obesity-related derivatives with cardio-renal-metabolic multimorbidity over two decades of follow-up [35]. The present study advances this evidence in two respects: it evaluates a harmonised eight-condition outcome spanning several organ systems rather than cardiometabolic phenotype, and it reproduces the association across three national settings using both cross-sectional and longitudinal designs.

The comparative analyses also clarify what can, and cannot, be concluded about the relative performance of TyG-WHtR. Combining TyG with an anthropometric measure is biologically and clinically plausible because the resulting index captures complementary dimensions of insulin resistance and adiposity [36, 37]. Previous studies have reported that TyG-WHtR, TyG-WC, TyG-BRI, TyG-ABSI, and other composite indices can outperform TyG alone for selected cardiovascular or multimorbidity outcomes [23–26, 38–43]. Nevertheless, recent head-to-head comparisons show that the leading index varies by population and outcome: TyG-ABSI performed well for cardiovascular disease in CHARLS [26], cumulative TyG-BRI was comparatively robust across CHARLS and ELSA [44], and TyG-WWI or TyG-CVAI showed stronger discrimination for cardiovascular-liver-metabolic multimorbidity [39]. In our data, TyG-WHtR had the highest area under the curve in NHANES (0.679), cross-sectional HRS (0.661), CHARLS (0.641), and ELSA (0.631). In prospective HRS, however, TyG-WC had a marginally higher AUC than TyG-WHtR, and the absolute differences among indices were small. TyG-WHtR should therefore be viewed as a simple and comparatively stable marker across cohorts, rather than as a universally superior predictor. Moreover, the cross-sectional ROC curves describe concurrent discrimination, not prediction of future disease, and the modest longitudinal AUCs indicate that TyG-WHtR is better suited to complement, rather than replace, established clinical risk assessment.

The nonlinear findings are a central contribution of this study. In CHARLS and ELSA, the estimated inflection points were close (5.92 and 5.80), and each 1-unit increase below these points was associated with 42% and 98% higher hazards of incident multimorbidity, respectively. In HRS, the corresponding point was higher (6.69), and the below-threshold association was weaker but remained statistically significant (hazard ratio 1.19; *P* = 0.048). Above the estimated points, none of the cohort-specific slopes was statistically significant. These results accord with growing evidence that relationships involving TyG-derived indices are not necessarily linear. Previous studies have described nonlinear associations of TyG-WHtR with cardiovascular disease [27] and of several TyG-related indices with cardiometabolic multimorbidity [38], while recent work has also identified nonlinear or saturating patterns for composite metabolic-adiposity exposures [44, 45]. In contrast, the earlier CHARLS study of TyG-WHtR and cardiometabolic multimorbidity reported a predominantly linear relationship [21]. Differences in disease composition, eligibility criteria, follow-up time, covariate structure, and the distribution of TyG-WHtR may explain these apparently divergent results.

The convergence of the cohort-specific inflection points at 5.92 in CHARLS, 5.80 in ELSA, and 6.69 in HRS represents an important finding of this study and suggests that a TyG-WHtR range of approximately 5.0–7.0 may constitute a potentially clinically informative risk-transition interval for incident multimorbidity. This interpretation is supported by previous studies reporting nonlinear TyG-WHtR associations with other cardiometabolic outcomes: an independent NHANES analysis identified an inflection point of 5.79 for hypertension [46], a prospective Chinese cohort reported a saturation threshold of 4.804 for incident cardiovascular disease [47], and the 15-year Kailuan cohort demonstrated a nonlinear association of TyG-WHtR with atherosclerotic cardiovascular disease and superior risk-stratification performance compared with TyG alone [48]. Taken together, these findings suggest that TyG-WHtR values approaching 5–7 may mark the upper boundary of a relatively steep risk-increase region and could provide a practical signal for more intensive metabolic assessment, multimorbidity screening, and early risk-factor management in middle-aged and older adults. Nevertheless, because the exact inflection point differed across cohorts and clinical outcomes, the proposed 5–7 range should be regarded as a provisional risk-alert interval rather than a definitive diagnostic or treatment threshold. Its reproducibility, calibration, and clinical utility should be further evaluated in large, multiethnic real-world populations with repeated TyG-WHtR measurements and independent external validation.

Several mechanisms may underlie the observed associations. The TyG component reflects fasting glucose and triglyceride disturbances that accompany insulin resistance, whereas WHtR captures abdominal adiposity relative to body size. Insulin resistance promotes compensatory hyperinsulinaemia, endothelial dysfunction, oxidative stress, dyslipidaemia, and activation of pro-inflammatory pathways [49, 50]. Visceral adipose tissue further contributes through increased release of free fatty acids and pro-inflammatory adipokines, impaired adiponectin signalling, and ectopic lipid deposition. These processes are relevant not only to diabetes and cardiovascular disease but also to pulmonary, musculoskeletal, and malignant disease pathways that contribute to generalised multimorbidity. WHtR may add value because normalising waist circumference for height reduces some of the body-size dependence of WC and may better represent central fat accumulation than BMI alone. TyG-WHtR therefore integrates two interacting components of a shared metabolic-inflammatory substrate.

From a clinical and public-health perspective, TyG-WHtR has practical advantages: its components are inexpensive, routinely measured, and interpretable across different care settings. It may help identify middle-aged and older adults who warrant a more comprehensive assessment of chronic disease burden and modifiable metabolic risk, particularly where insulin measurements or advanced imaging are unavailable. The findings do not imply that TyG-WHtR should be used as a stand-alone screening test. Rather, it could be evaluated as an adjunct to age, blood pressure, comorbidity history, lifestyle factors, and standard laboratory measures. Future work should determine whether adding TyG-WHtR improves calibration, net benefit, and clinical decisions beyond these established variables, and whether reductions in TyG-WHtR over time translate into lower multimorbidity risk.

This study integrated five analytic samples from four nationally representative cohorts across China, England, and the United States, combining cross-sectional replication with prospective analyses. Use of the same eight-condition definition improved cross-cohort comparability. TyG-WHtR was evaluated continuously and categorically, compared with five alternative TyG-related indices, and examined using nonlinear and threshold analyses. Robustness was further assessed using lagged follow-up and alternative multimorbidity definitions.Several limitations should be acknowledged. The observational design precludes causal inference, and residual confounding remains possible. Reliance on self-reported diagnoses may have introduced misclassification, while the harmonised definition did not capture disease severity or clustering. Single baseline measurements, loss to follow-up, and competing mortality may also have affected the estimates. The smaller prospective HRS sample reduced precision, and the subgroup findings were exploratory and should be interpreted cautiously because of multiple testing and small sample sizes in several strata. Finally, discrimination was modest, calibration and clinical utility were not evaluated, and the inflection points were internally derived. Therefore, the proposed TyG-WHtR range of approximately 5–7 should be considered a candidate risk-alert interval requiring validation in independent, real-world populations.

## Conclusion

Across five analytic samples from four national studies, higher TyG-WHtR was associated with prevalent multimorbidity in both cross-sectional samples and with incident multimorbidity in quartile-based analyses across all three prospective cohorts, although the continuous association was not statistically significant in prospective HRS. TyG-WHtR generally showed the strongest or near-strongest discrimination among the examined TyG-related anthropometric indices, but its absolute discriminatory performance was modest and its advantage was not uniform across cohorts. The cohort-specific inflection points, ranging from 5.80 to 6.69, support a provisional candidate risk-alert interval of approximately 5–7. This interval is hypothesis-generating, requires independent external validation, and should not be interpreted as a diagnostic or treatment threshold. TyG-WHtR may serve as a simple, low-cost adjunctive marker for further metabolic assessment and multimorbidity screening.

## Supplementary Information

Below is the link to the electronic supplementary material.

Supplementary Material 1.

Supplementary Material 2.

## Data Availability

The datasets analyzed in this study are available from the official repositories of NHANES, CHARLS, ELSA, and HRS, subject to the registration and data-use requirements of the respective studies.

## Abbreviations

**ABSI**: A body shape index
**AUC**: Area under the curve
**BMI**: Body mass index
**BRI**: Body roundness index
**CHARLS**: China health and retirement longitudinal study
**CI**: Confidence interval
**CRP**: C-reactive protein
**ELSA**: English longitudinal study of ageing
**HR**: Hazard ratio
**HRS**: Health and retirement study
**IR**: Insulin resistance
**NHANES**: National health and nutrition examination survey
**OR**: Odds ratio
**RCS**: Restricted cubic spline
**ROC**: Receiver operating characteristic
**TyG**: Triglyceride-glucose index
**TyG-ABSI**: Triglyceride-glucose–a body shape index
**TyG-BMI**: Triglyceride-glucose–body mass index
**TyG-BRI**: Triglyceride-glucose–body roundness index
**TyG-WC**: Triglyceride-glucose–waist circumference
**TyG-WHtR**: Triglyceride-glucose waist-to-height ratio
**TyG-WWI**: Triglyceride-glucose–weight-adjusted waist index
**WC**: Waist circumference
**WHtR**: Waist-to-height ratio
**WWI**: Weight-adjusted waist index
