# Supplementary material for: Triglyceride-glucose waist-to-height ratio in relation to prevalent and incident multimorbidity: cross-sectional and prospective evidence from four national studies
Source: Cardiovasc Diabetol. 2026 Aug 24;25:286. doi: 10.1186/s12933-026-03320-y (PMC13602531; doi:10.1186/s12933-026-03320-y)
Supplement: Supplementary file 1 [file 12933_2026_3320_MOESM1_ESM.docx]

**Figure S1. Participant-selection flowcharts for the expanded 14-condition sensitivity analyses in CHARLS and ELSA.(A) CHARLS cohort; (B) ELSA cohort.**

**Figure S2. Restricted cubic spline association between TyG-WHtR and incident multimorbidity based on the 14-condition definition in CHARLS.**

The solid purple line represents the adjusted hazard ratio, the shaded area and blue dashed lines represent the 95% confidence interval, and the histogram shows the distribution of TyG-WHtR. The horizontal dashed line indicates an HR of 1.00.

**Figure S3. Restricted cubic spline association between TyG-WHtR and incident multimorbidity based on the 14-condition definition in ELSA.**

The solid purple line represents the adjusted hazard ratio, the shaded area and blue dashed lines represent the 95% confidence interval, and the histogram shows the distribution of TyG-WHtR. The horizontal dashed line indicates an HR of 1.00.
