# Supplementary material for: Triglyceride-glucose waist-to-height ratio in relation to prevalent and incident multimorbidity: cross-sectional and prospective evidence from four national studies
Source: Cardiovasc Diabetol. 2026 Aug 24;25:286. doi: 10.1186/s12933-026-03320-y (PMC13602531; doi:10.1186/s12933-026-03320-y)
Supplement: Supplementary file 2 [file 12933_2026_3320_MOESM2_ESM.docx]

## **Table S1. Definitions and formulas of the TyG-related indices evaluated in the study**

| **Index** | **Formula** | **Required units** |
| --- | --- | --- |
| BMI | Weight / height² | Weight in kg; height in m |
| WC | Waist circumference | cm |
| WHtR | WC / height | WC and height in the same units |
| BRI | 364.2 − 365.5 × √{1 − [WC / (2π)]² / (height / 2)²} | WC and height in the same units |
| ABSI | WC / (BMI²/³ × height¹/²) | WC and height in m; BMI in kg/m² |
| WWI | WC / √weight | WC in cm; weight in kg |
| TyG-BMI | TyG × BMI | — |
| TyG-WC | TyG × WC | WC in cm |
| TyG-WHtR | TyG × WHtR | — |
| TyG-BRI | TyG × BRI | — |
| TyG-ABSI | TyG × ABSI | — |
| TyG-WWI | TyG × WWI | — |

Abbreviations: ABSI, a body shape index; BMI, body mass index; BRI, body roundness index; FBG, fasting blood glucose; TG, fasting triglycerides; TyG, triglyceride-glucose index; WC, waist circumference; WHtR, waist-to-height ratio; WWI, weight-adjusted waist index.

Note: Glucose and triglyceride concentrations recorded in mmol/L were converted to mg/dL using factors of 18.018 and 88.57, respectively, before calculation of TyG.

**Table S2. Harmonized ascertainment of the eight chronic conditions included in the primary definition of multimorbidity**

| **Chronic condition** | **Harmonized operational definition** | **NHANES** | **CHARLS** | **ELSA** | **HRS** |
| --- | --- | --- | --- | --- | --- |
| Hypertension | Any of: previous physician diagnosis; current antihypertensive medication; or mean SBP ≥140 mmHg or DBP ≥90 mmHg. | Questionnaire diagnosis and antihypertensive medication, supplemented by the mean of available examination BP readings. | Questionnaire diagnosis or antihypertensive treatment, supplemented by the mean of three seated BP readings. | Doctor-diagnosed hypertension or antihypertensive treatment, supplemented by nurse-measured BP. | Doctor-diagnosed hypertension or antihypertensive treatment, supplemented by HRS physical-measure BP readings. |
| Dyslipidemia | Any of: previous physician diagnosis/high cholesterol; current lipid-lowering medication; TC ≥240 mg/dL, LDL-C ≥160 mg/dL, HDL-C <40 mg/dL, or TG ≥200 mg/dL. | Questionnaire diagnosis and lipid-lowering medication, supplemented by the NHANES lipid panel. | Questionnaire diagnosis or lipid-lowering treatment, supplemented by baseline biomarker lipid measurements. | Doctor-diagnosed high cholesterol or lipid-lowering treatment, supplemented by nurse-collected lipid measurements. | Doctor-diagnosed high cholesterol or lipid-lowering treatment, supplemented by the 2016 VBS lipid panel. |
| Diabetes | Any of: previous physician diagnosis; current glucose-lowering medication or insulin; or fasting plasma glucose ≥126 mg/dL. | Questionnaire diagnosis and diabetes medication/insulin, supplemented by fasting plasma glucose. | Questionnaire diagnosis or diabetes treatment, supplemented by fasting plasma glucose. | Doctor-diagnosed diabetes or glucose-lowering treatment, supplemented by fasting plasma glucose. | Doctor-diagnosed diabetes or glucose-lowering treatment, supplemented by 2016 VBS fasting glucose. |
| Cancer | Previous physician diagnosis of cancer or a malignant tumor. | Physician/health-professional diagnosis of cancer or malignancy. | Physician diagnosis of cancer or malignant tumor. | Doctor diagnosis of cancer or malignant tumor. | Doctor diagnosis of cancer or malignant tumor. |
| Chronic lung disease | Previous physician diagnosis of chronic lung disease, chronic bronchitis, emphysema, or chronic obstructive pulmonary disease, using the closest available item. | Physician diagnosis of emphysema, chronic bronchitis, or the corresponding chronic lung condition. | Physician diagnosis of chronic lung disease, including chronic bronchitis or emphysema. | Doctor diagnosis of chronic lung disease. | Doctor diagnosis of chronic lung disease, including chronic bronchitis or emphysema. |
| Heart disease | At least one physician-diagnosed cardiac condition: coronary heart disease, myocardial infarction, angina, congestive heart failure, or another heart condition. | Any corresponding physician-diagnosed coronary heart disease, myocardial infarction, angina, or congestive heart failure item. | Physician-diagnosed heart disease. | Any corresponding doctor-diagnosed coronary heart disease, myocardial infarction, angina, or heart failure item. | Any corresponding doctor-diagnosed myocardial infarction, angina, congestive heart failure, or other heart condition. |
| Stroke | Previous physician diagnosis of stroke. | Physician/health-professional diagnosis of stroke. | Physician diagnosis of stroke. | Doctor diagnosis of stroke. | Doctor diagnosis of stroke. |
| Arthritis | Previous physician diagnosis of arthritis or rheumatism. | Physician/health-professional diagnosis of arthritis. | Physician diagnosis of arthritis or rheumatism. | Doctor diagnosis of arthritis. | Doctor diagnosis of arthritis or rheumatism. |

Abbreviations: BP, blood pressure; CHARLS, China Health and Retirement Longitudinal Study; DBP, diastolic blood pressure; ELSA, English Longitudinal Study of Ageing; HDL-C, high-density lipoprotein cholesterol; HRS, Health and Retirement Study; LDL-C, low-density lipoprotein cholesterol; NHANES, National Health and Nutrition Examination Survey; SBP, systolic blood pressure; TC, total cholesterol; TG, triglycerides.

**Table S3. Cohort-specific composition of the broader 14-condition definition of multimorbidity used in supplementary analyses**

| **No.** | **CHARLS** | **ELSA** |
| --- | --- | --- |
| 1 | Hypertension | Hypertension |
| 2 | Dyslipidemia | Dyslipidemia |
| 3 | Diabetes | Diabetes |
| 4 | Heart disease | Heart disease |
| 5 | Stroke | Stroke |
| 6 | Cancer | Cancer |
| 7 | Chronic lung disease | Chronic lung disease |
| 8 | Digestive system disease | Arthritis |
| 9 | Liver disease | Asthma |
| 10 | Kidney disease | Emotional or psychiatric problems |
| 11 | Asthma | Memory-related disease |
| 12 | Arthritis | Osteoporosis |
| 13 | Memory-related disease | Parkinson’s disease |
| 14 | Emotional or psychiatric disorders | Cataracts |

Note: The expanded outcome was defined as at least two of the 14 cohort-specific conditions. Its composition differed between CHARLS and ELSA because of questionnaire and data availability; it was therefore used only in sensitivity analyses.

**Table S4. Definitions and harmonization of covariates across the study cohorts**

| **Covariate** | **Harmonized definition and coding** | **Cohort-specific implementation** |
| --- | --- | --- |
| Age | Age at the corresponding baseline assessment, analyzed continuously in years | Derived from the baseline interview or examination in NHANES, CHARLS, ELSA, and HRS |
| Sex | Male or female | Based on the sex variable recorded in each cohort |
| Education | elementary, high school, and university levels | Original educational qualifications were mapped to a three-level cohort-specific classification according to the highest level completed |
| Marital status | Married versus other | Participants currently recorded as married were classified as married; all non-married categories, including never married, separated, divorced, and widowed, were combined as other |
| Smoking status | Cohort-specific smoking status | NHANES: never, former, or current; CHARLS, ELSA, and HRS: current smoking, No or Yes |
| drinking status | Cohort-specific alcohol-consumption status | NHANES: never, former, or current; CHARLS, ELSA, and HRS: current drinking, No or Yes |
| Physical activity | Low, Medium, or high | NHANES: tertiles of the available activity measure; HRS: 0 = low, 1-2 = moderate, and 3-4 = high; CHARLS and ELSA: cohort-specific frequency and intensity categories |
| Household income | Low, Medium, or high | Cohort-specific categories or distribution-based groups were used because absolute income was not comparable across countries and survey years |
| Health insurance | Uninsured or insured | Closest available measure of current health insurance or healthcare coverage in each cohort |
| C-reactive protein | Continuous variable | CRP concentrations were harmonized to a common unit before analysis |
| Hemoglobin | Continuous variable | Hemoglobin concentrations were harmonized to a common unit before analysis |
| Race/ethnicity | Cohort-specific racial/ethnic categories | Included only in NHANES; categories were defined using the NHANES race/ethnicity variable |
| Residence | Rural versus urban | Included only in CHARLS according to the cohort-specific residence variable |

Abbreviations: CHARLS, China Health and Retirement Longitudinal Study; CRP, C-reactive protein; ELSA, English Longitudinal Study of Ageing; HRS, Health and Retirement Study; NHANES, National Health and Nutrition Examination Survey.

Note: Covariates were harmonized conceptually while retaining cohort-specific coding. Race/ethnicity was additionally included only in NHANES and place of residence only in CHARLS.

**Table S5. Baseline characteristics of participants in ELSA according to incident multimorbidity status**

| **Characteristic** | **Total (n = 1310)** | **No incident multimorbidity (n = 585)** | **Incident multimorbidity (n = 725)** | **P** |
| --- | --- | --- | --- | --- |
| Age, years | 61.24 ± 6.37 | 60.46 ± 6.19 | 61.87 ± 6.44 | <.001 |
| Weight, kg | 75.13 ± 17.65 | 73.04 ± 16.04 | 76.82 ± 18.69 | <.001 |
| CRP, mg/L | 3.46 ± 5.47 | 3.18 ± 5.82 | 3.68 ± 5.15 | 0.097 |
| Hemoglobin, g/dL | 13.83 ± 2.74 | 13.78 ± 2.67 | 13.87 ± 2.80 | 0.559 |
| Sex, n (%) |  |  |  | 0.918 |
| Female | 719 (54.89) | 322 (55.04) | 397 (54.76) |  |
| Male | 591 (45.11) | 263 (44.96) | 328 (45.24) |  |
| Education, n (%) |  |  |  | <.001 |
| elementary school | 388 (29.62) | 142 (24.27) | 246 (33.93) |  |
| high school | 322 (24.58) | 157 (26.84) | 165 (22.76) |  |
| university | 600 (45.80) | 286 (48.89) | 314 (43.31) |  |
| Marital status, n (%) |  |  |  | 0.176 |
| Married | 991 (75.65) | 453 (77.44) | 538 (74.21) |  |
| Other | 319 (24.35) | 132 (22.56) | 187 (25.79) |  |
| Health insurance , n (%) |  |  |  | 0.140 |
| No | 1058 (80.76) | 462 (78.97) | 596 (82.21) |  |
| Yes | 252 (19.24) | 123 (21.03) | 129 (17.79) |  |
| Smoking status, n (%) |  |  |  | 0.998 |
| No | 1160 (88.55) | 518 (88.55) | 642 (88.55) |  |
| Yes | 150 (11.45) | 67 (11.45) | 83 (11.45) |  |
| Drinking status, n (%) |  |  |  | 0.053 |
| No | 74 (5.65) | 25 (4.27) | 49 (6.76) |  |
| Yes | 1236 (94.35) | 560 (95.73) | 676 (93.24) |  |
| Physical activity, n (%) |  |  |  | 0.340 |
| High | 978 (74.66) | 448 (76.58) | 530 (73.10) |  |
| Low | 73 (5.57) | 29 (4.96) | 44 (6.07) |  |
| Moderate | 259 (19.77) | 108 (18.46) | 151 (20.83) |  |
| Household income, n (%) |  |  |  | 0.013 |
| High | 438 (33.44) | 220 (37.61) | 218 (30.07) |  |
| Low | 732 (55.88) | 303 (51.79) | 429 (59.17) |  |
| Middle | 140 (10.69) | 62 (10.60) | 78 (10.76) |  |

Values are mean ± standard deviation or n (%). P values were calculated using the independent-samples t test, chi-square test, or Fisher exact test, as appropriate. Percentages are based on available observations.

**Table S6. Baseline characteristics of participants in HRS according to incident multimorbidity status**

| **Characteristic** | **Total (n = 971)** | **No incident multimorbidity (n = 688)** | **Incident multimorbidity (n = 283)** | **P** |
| --- | --- | --- | --- | --- |
| Age, years | 63.85 ± 8.23 | 63.45 ± 8.03 | 64.82 ± 8.62 | 0.019 |
| Weight, kg | 79.32 ± 17.16 | 78.54 ± 16.96 | 81.27 ± 17.51 | 0.026 |
| Hemoglobin, g/dL | 13.86 ± 1.35 | 13.82 ± 1.29 | 13.95 ± 1.50 | 0.198 |
| CRP, mg/L | 3.82 ± 7.25 | 3.68 ± 7.20 | 4.17 ± 7.36 | 0.341 |
| Sex, n (%) |  |  |  | 0.047 |
| Female | 606 (62.41) | 443 (64.39) | 163 (57.60) |  |
| Male | 365 (37.59) | 245 (35.61) | 120 (42.40) |  |
| Education, n (%) |  |  |  | 0.040 |
| elementary school | 114 (11.74) | 71 (10.32) | 43 (15.19) |  |
| high school | 548 (56.44) | 384 (55.81) | 164 (57.95) |  |
| Higher | 309 (31.82) | 233 (33.87) | 76 (26.86) |  |
| Marital status, n (%) |  |  |  | 0.503 |
| Married | 667 (68.69) | 477 (69.33) | 190 (67.14) |  |
| Other | 304 (31.31) | 211 (30.67) | 93 (32.86) |  |
| Health insurance, n (%) |  |  |  | 0.008 |
| No | 612 (63.03) | 452(65.70) | 160 (56.54) |  |
| Yes | 359 (36.97) | 236 (34.40) | 123 (43.46) |  |
| Smoking status, n (%) |  |  |  | 0.007 |
| No | 851 (87.64) | 616 (89.53) | 235 (83.04) |  |
| Yes | 120(12.36) | 72(10.47) | 48 (16.96) |  |
| Drinking status, n (%) |  |  |  | 0.031 |
| No | 318 (32.75) | 211 (30.67) | 107 (37.81) |  |
| Yes | 653 (67.25) | 477 (69.33) | 176 (62.19) |  |
| Physical activity, n (%) |  |  |  | 0.002 |
| High | 590 (60.76) | 440 (63.95) | 150 (53.00) |  |
| Low | 104 (10.71) | 62 (9.01) | 42 (14.84) |  |
| Moderate | 277(28.53) | 186 (27.03) | 91(32.15) |  |
| Household income, n (%) |  |  |  | 0.166 |
| High | 322 (33.16) | 238 (34.59) | 84 (29.68) |  |
| Low | 459 (47.27) | 312 (45.35) | 147 (51.94) |  |
| Middle | 190 (19.57) | 138 (20.06) | 52 (18.37) |  |

Values are mean ± standard deviation or n (%). P values were calculated using the independent-samples t test, chi-square test, or Fisher exact test, as appropriate. Percentages are based on available observations; denominators may vary because of item-level missingness.

**Table S7. Sensitivity analyses of the association between TyG-WHtR quartiles and incident multimorbidity in ELSA**

| **Analysis** | **N** | **Q2 vs Q1, HR (95% CI)** | **Q3 vs Q1, HR (95% CI)** | **Q4 vs Q1, HR (95% CI)** | ***P* for trend** |
| --- | --- | --- | --- | --- | --- |
| Primary analysis: ≥2 of 8 harmonized conditions | 1,310 | 1.25 (0.98–1.58) | 1.67 (1.31–2.11) | 1.67 (1.30–2.15) | <0.001 |
| Excluding incident events within the first 2 years | 1,094 | 1.18 (0.90–1.55) | 1.52 (1.15–2.01) | 1.59 (1.18–2.13) | <0.001 |
| Stricter definition: ≥3 of 8 harmonized conditions | 1,310 | 1.12 (0.81–1.55) | 1.44 (1.05–1.97) | 1.83 (1.32–2.53) | <0.001 |
| Expanded definition: ≥2 of 14 ELSA-specific conditions | 1,278 | 1.14 (0.93–1.40) | 1.39 (1.13–1.70) | 1.33 (1.09–1.61) | <0.001 |

Values are HRs with 95% CIs. Q1 was the reference. Model 3 adjusted for age, sex, education, marital status, health insurance , physical activity, household income, smoking status,drinking status, CRP, and hemoglobin. The primary, lagged, and stricter-definition analyses used the eight harmonized conditions; the expanded analysis used 14 ELSA-specific conditions. Abbreviations: CI, confidence interval; CRP, C-reactive protein; ELSA, English Longitudinal Study of Ageing; HR, hazard ratio; Q, quartile; TyG-WHtR, triglyceride-glucose waist-to-height ratio index.
